# *N*-Acetylcysteine Added to Local Anesthesia Reduces Scar Area and Width in Early Wound Healing—An Animal Model Study

**DOI:** 10.3390/ijms22147549

**Published:** 2021-07-14

**Authors:** Wiktor Paskal, Adriana M. Paskal, Piotr Pietruski, Albert Stachura, Kacper Pełka, Alan E. Woessner, Kyle P. Quinn, Michał Kopka, Ryszard Galus, Jarosław Wejman, Paweł Włodarski

**Affiliations:** 1 Department of Methodology, Medial University of Warsaw, 02-091 Warsaw, Poland; adriana.paskal@gmail.com (A.M.P.); albert.stachura@wum.edu.pl (A.S.); kacper.pelka@wum.edu.pl (K.P.); m_kopka@wp.pl (M.K.); pawel.wlodarski@wum.edu.pl (P.W.); 2 Department of Replantation and Reconstructive Surgery, Centre of Postgraduate Medical Education, Gruca Teaching Hospital, 05-400 Otwock, Poland; pietruski.piotr@gmail.com; 3 Doctoral School, Medical University of Warsaw, 02-091 Warsaw, Poland; 4 Department of Biomedical Engineering, University of Arkansas, Fayetteville, AR 72701, USA; aewoessn@email.uark.edu (A.E.W.); kpquinn@uark.edu (K.P.Q.); 5 Department of Histology and Embryology, Medical University of Warsaw, 02-091 Warsaw, Poland; ryszard.galus@wum.edu.pl; 6 Department of Pathology, Centre for Postgraduate Education, 00-416 Warsaw, Poland; jarwej@poczta.fm

**Keywords:** wound healing, skin, *N*-Acetylcysteine, pretreatment, rat, local anesthesia additive, incision, surgical

## Abstract

The aim of the study was to evaluate if a pre-incisional *N*-acetylcysteine (NAC) treatment altered the process of wound healing in a rat model. The dorsal skin of 24 Sprague-Dawley rats was incised in six locations. Before the incisions were made, skin was injected either with lidocaine and epinephrine (one side) or with these agents supplemented with 0.015%, 0.03%, or 0.045% NAC (contralaterally). Photographic documentation of the wound healing process was made at 11 time points. Rats were sacrificed 3, 7, 14, or 60 days after incision to excise scars for histological analysis. They included: Abramov scale scoring, histomorphometry analysis, and collagen fiber arrangement assessment. Skin pretreated with 0.03% NAC produced the shortest scars at all analyzed time points, though this result was statistically insignificant. At this NAC concentration the scars had smaller areas on the third day and were narrower on the day 4 compared with all the other groups (*p* < 0.05). On day 7, at the same concentration of NAC, the scars had a higher superficial concentration index (*p* = 0.03) and larger dermal proliferation area (*p* = 0.04). NAC addition to pre-incisional anesthetic solution decreased wound size and width at an early stage of scar formation at all concentrations; however, with optimal results at 0.03% concentration.

## 1. Introduction

Wound healing is a complex process, comprised of consecutive phases: hemostasis, inflammation, proliferation, and remodeling [1]. The transition from inflammation to proliferation is crucial for prompting adequate tissue regeneration. Novel therapeutic approaches focus on improving this step by administering substances, which often alleviate the inflammatory processes [2]. These interventions, however, are usually applied after the injury has occurred.

Planned surgical incisions allow implementing pro-regenerative interventions prior to wound creation. Local anesthetics, such as lidocaine with vasoconstricting components, are applied before incising tissues [3]. Supplementing the anesthetic solution with an adjuvant enhancing tissue regeneration may improve wound healing. Currently, there is scarce data on research incorporating local, pre-incisional strategies in wound management [4]. Topical vitamin E pre-incisional ointments were proven to enhance wound healing in children [5].

*N*-Acetylcysteine (NAC) is a widely used cytoprotective pharmaceutical. It directly reduces the level of reactive oxygen species (ROS) and increases the synthesis of glutathione (endogenous antioxidant), exerting an overall antioxidative effect on tissues [6,7]. Both mechanisms may contribute to limiting the extent of the inflammation that occurs during the early stages of wound healing.

Previous studies showed that NAC improves wound breaking strength, epithelialization, and healing in rat and murine wound models [8,9,10,11,12]. Previous studies showed that NAC significantly lowers malondialdehyde levels and increases glutathione levels in wounded tissues [8]. In vitro experiments proved that NAC promotes *MMP-1* (Matrix metalloproteinase 1) expression via the *PI3K* (phosphatidylinositol 3-kinase) and *Stat3* (signal transducer and activator of transcription 3) signaling pathways. This finding was associated with increased growth (significant increase in the number of viable cells at 1.0 mM NAC) and a dose-dependent increase in migration of human fibroblasts when treated with *N*-acetylcysteine in a scratch wound healing assay [9]. NAC at lower concentrations stimulates keratinocyte proliferation in vitro, but acts conversely above a concentration of 2 mM. It also promotes cellular differentiation, formation of intercellular junctions, and upregulates genes *p53*, *E-cadherin*, and *HSP27* (heat shock protein 27) [13].

In vivo studies demonstrated that NAC significantly improved wound healing rate and wound histology [10,11,12]. Gomez-Aparicio et al. used NAC enriched hydrogel and improved the wound closure rate, early wound size, and re-epithelization, and achieved more organized collagen deposits in murine wounds [11]. Similar observations were reported by Oguz et al., who compared topical NAC or dexpanthenol and sham ointment treatment. Moreover, they noted increased angiogenesis in the NAC group and decreased fibrosis in both experimental groups, as well as similar wound healing rate vs. control (*p* < 0.05) [12]. In the case of bur wounds, 3% NAC dressing decreased oxidative stress in skin tissue and improved re-epithelization [9]. Diabetic wounds also benefited from topical NAC administration. Ozkaya et al. [10] reported decreased inflammation and oxidative stress and improved re-epithelization, as well as higher wound breakage strength, according to Aktunc et al. [8].

To date, all studies on NAC usage in wound healing have focused exclusively on the general administration or topical use of NAC on an existing wound. Moreover, the observation period in the above-mentioned studies lasted not more than 14 days, thus providing no information on the effects of NAC on the later, remodeling phase [8,9,10,11,12]. NAC has never been investigated as a healing modulating agent used prior to wound creation.

In this study, we enriched an anesthetic solution with NAC and assessed how the pre-incisional administration of this solution affected the healing process in a rat skin deep wound model. We did observations for 60 days and analyzed the impact of NAC on all wound healing phases. We performed a planimetric evaluation of photographed wounds and a histomorphometric analysis of the tissues at four time points: on the 3rd, 7th, 14th, and 60th day of the experiment. In addition to that, we studied the distribution and alignment of collagen fibers.

## 2. Results

**Figure 1:**
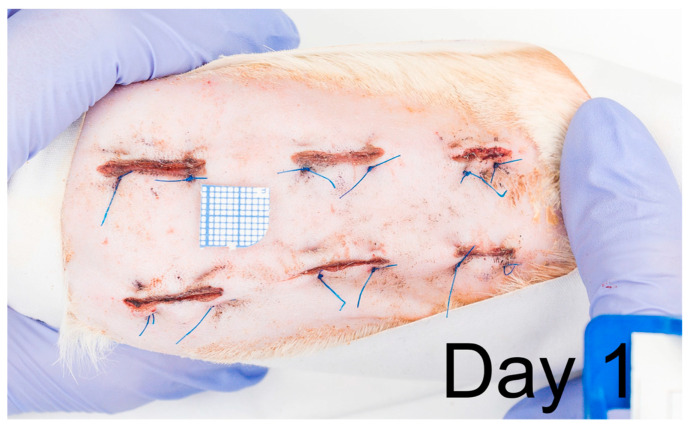
Video presents wound healing process captured with 11 time points of 60-days observation of a representative rat.

All animals completed the study. Each analyzed section was tested (ANOVA or Kruskal–Wallis test) for statistically significant differences between experimental groups (NAC15, NAC30, and NAC45). In the case of no differences, results were grouped (named gNAC) and compared with the control group (CONT). Figure 1 shows a representative wound healing process with one animal in the study.

### 2.1. Wound and Scar Planimetric Analysis Based on Photographic Documentation

**Figure 2:**
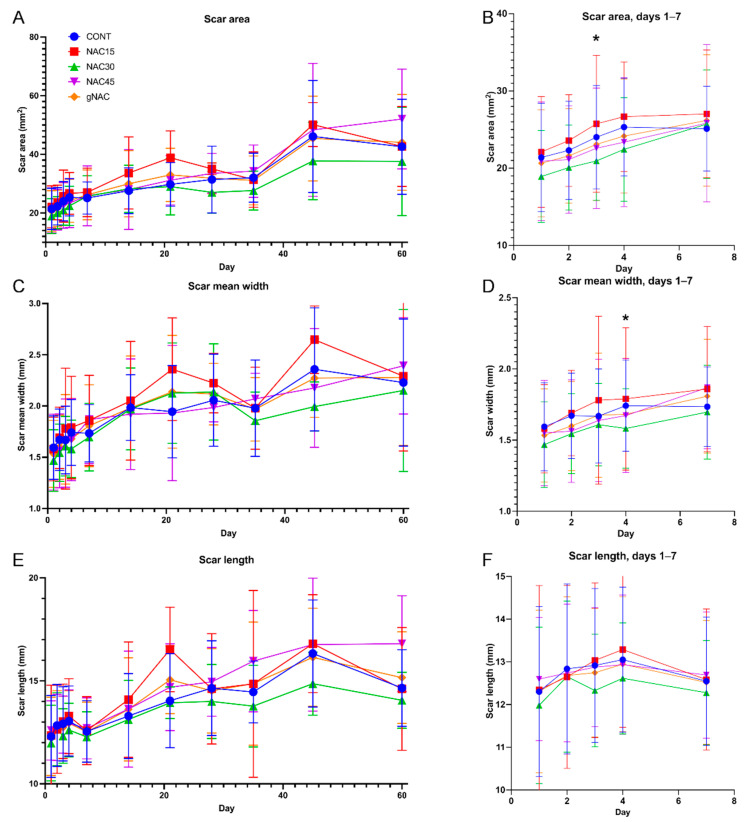
Graph represents results of planimetric measurements of photographed scars. (**A**) scar areas at days 1–60, (**B**) scar lengths at days 1–7, (**C**) scar mean width at days 1–60, (**D**) scar mean width at days 1–7, (**E**) scar lengths at day 1–60, (**F**) scar lengths at day 1–7. Mean results ± SD. * *p* < 0.05 (NAC30 vs. CONT).

The scar areas in the NAC30 group were smaller on the 3rd day of observation compared with the control group (*p* = 0.02, Figure 2B). The mean values of scar areas in the NAC30 group were lower than in any other group at all time points; however, not reaching statistical significance (Figure 2A).

The mean values of the scar width were also lower in the NAC30 group, compared with the remaining groups (*p* = 0.02), on the 4th day of observation. This trend was upheld throughout the study period, though without statistical significance (Figure 2C,D).

There were no statistically significant differences (ANOVA and post-hoc Turkey’s tests) in the scar lengths between the NAC groups compared with the control group. The mean lowest values of this parameter were noted in the NAC30 group at all time points; however, statistically insignificant. In the case of the NAC15 and NAC45 groups, on average, the scars were longer than in the CONT group (*p* > 0.05, Figure 2E,F).

### 2.2. Histological Assessment of Scars

**Figure 3:**
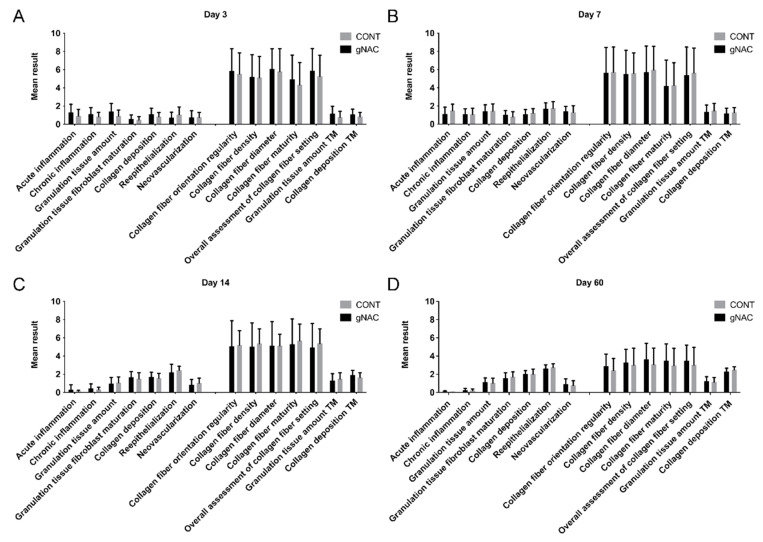
Graph presents results of histological analysis with Abramov scale. Results of groups NAC15, NAC30, and NAC45 collectively analyzed as gNAC. (**A**) Day 3, (**B**) day 7, (**C**) day 14, (**D**) day 60. Mean values are shown. Error bars are SD. TM—Masson’s Trichrome stained images.

**Figure 4:**
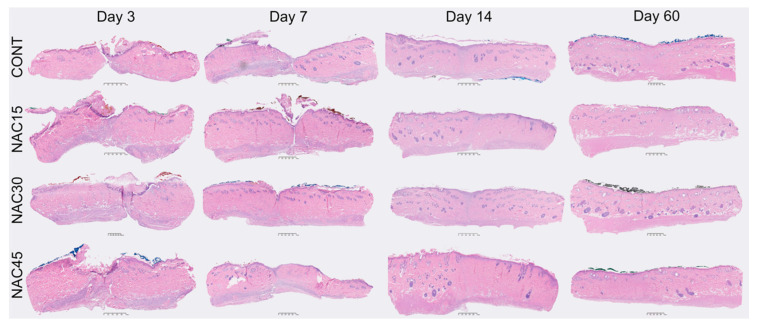
HE sections of representative samples from each group (NAC15, NAC30, NAC45, and CONT) at each harvest point. 1mm scale bar provided under each sample.

There were no statistically significant differences (ANOVA, *p* > 0.05) among the NAC15, NAC30, and NAC45 mean values for any of the variables at all-time points. Further analyses focused on the comparison between the gNAC and CONT groups. Results are summarized in Figure 3 and followed by representative images of HE stained slides from each experimental group at the four time points (Figure 4).

We observed a higher number of inflammatory cells, typical for acute and chronic inflammation, in the gNAC group compared with the control group (*p* = 0.14; *p* = 0.15) on the 3rd day. Conversely, on the 7th day, the acute inflammation was higher in CONT group than in the gNAC group (*p* = 0.13). At all the other time points, the intensity of both acute and chronic inflammatory processes was comparable.

The granulation process was assessed with both HE and MT staining. The granulation was more intense in gNAC than in the control group (HE, *p* = 0.06; MT *p* = 0.15) on the 3rd day. At all the later time points, no significant differences between gNAC and CONT were noted. The mean level of fibroblast maturity was higher in gNAC vs. CONT on days 3, 7, and 14. However, on the 60th day, it was higher in the control group (*p* > 0.05).

The parameters describing the size of collagen fibers deposits were assessed in HE- and MT-stained samples. On the 3rd day, there were more collagen fibers in gNAC than in the control group (*p* = 0.15; *p* = 0.19). However, at later time points the difference was less obvious (*p* > 0.05).

MT staining allowed assessing the collagen fibers’ orientation, morphology, and maturity. There were no statistically significant differences in these parameters between the studied groups, at all time points. On the days following the incision, a disorderly arrangement and immaturity of collagen fibers gradually decreased in both (gNAC and CONT) groups. The overall assessment of fiber orientation and morphology on days 3 and 60 showed insignificant differences; a more consistent collagen organization in the gNAC group (*p* = 0.43; *p* = 0.45) and less consistent on days 7 and 14 (*p* = 0.81; *p* = 0.64).

In the HE-stained samples, the levels of epithelialization and neovascularization were assessed. The epithelialization was less advanced in the gNAC group than in the control group (*p* > 0.3) at all time points. However, the differences between the mean values of this parameter in both groups became less explicit over time. No statistically significant differences in the number of newly formed blood vessels were noted between all the groups, at all time points.

There were no statistically significant differences in the wound closure rate, i.e., approximation of the wound edges, between the gNAC and the control group at any chosen time point (*p* > 0.05).

### 2.3. Histomorphometry

Histological slides from the 3rd day after incision were insufficient for a thorough and repeatable measurement analysis of the chosen parameters due to substantial artifacts (e.g., rupture of scars).

**Figure 5:**
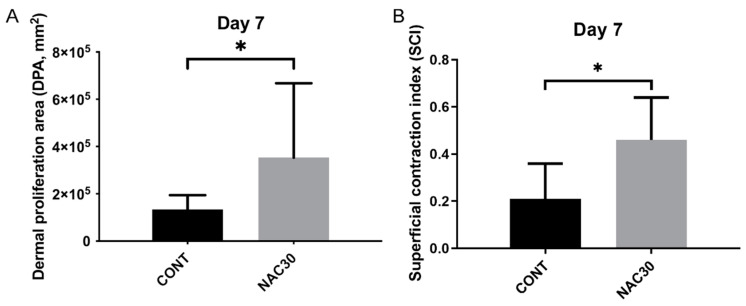
Graph summarizing differences in dermal proliferation area (**A**) and superficial contraction index (**B**) between NAC30 and CONT at day 7. Data expressed as mean ± SD, * *p* < 0.05.

We searched for statistically significant differences of all the morphometric parameters in the HE- and MT-stained samples. The groups treated with various NAC concentrations (NAC15, 30, 45) were compared. The mean values of all the parameters did not differ significantly (ANOVA, *p* > 0.05) between the groups at all time points, apart from on the 7th day (ANOVA, *p* > 0.05) (Table 1, Figure 5).

DPAs (dermal proliferation areas) were significantly larger in the group pre-treated with 0.03% NAC concentration (*p* = 0.04, Figure 5. All the other variables, describing the scar size, did not vary significantly between all the NAC groups (*p* > 0.05). The thickness of the newly-formed epidermis was larger in both the gNAC and NAC30 groups compared with the control group. The distance between the wound edges was, on average, smaller in the gNAC and NAC30 groups than in the control group.

The SCI (superficial contraction index) was significantly higher in the NAC30 group compared with the control (*p* < 0.03, Figure 5). The administration of NAC, regardless of the concentration, elevated the value of SCI on the 7th day; however, without statistical significance (*p* = 0.07). All other parameters describing the wound healing process did not vary significantly between the studied groups and the control group. The general healing index (GHI) on the 7th day was higher in the NAC30 group than in the control group (*p* = 0.55).

We did not find any statistically significant differences in the basic (D, DPA, EPI, L, N, NEO, S, T, H, B) parameters or in the wound healing indexes between the NAC groups and the control group on days 14 and 60.

### 2.4. Collagen Fiber Organization

**Figure 6:**
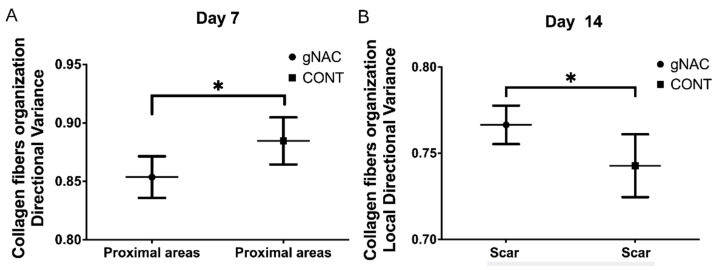
Graph represents directional variance distribution of collagen fibers in proximal scar areas (**A**) and scars (**B**) on the 7th and 14th days, respectively, in gNAC vs. CONT groups. Values expressed as mean ± SD, * *p* < 0.05.

**Figure 7:**
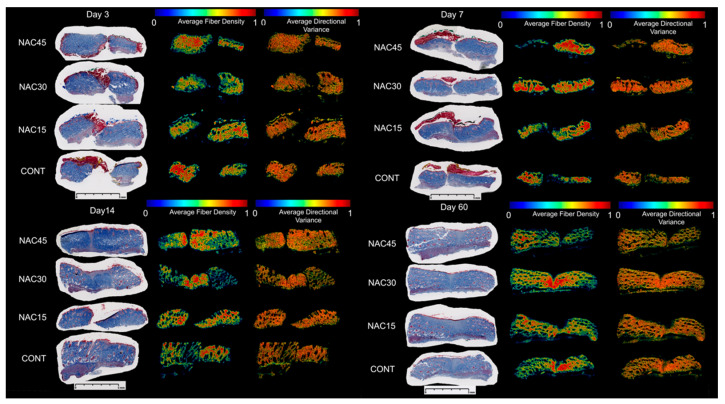
Representative results of automated collagen fiber arrangement analysis by Quinn et al. Heatmaps of collagen fiber density and average directional variance are presented along with Trichrome Masson’s staining of each group (NAC15, NAC30, NAC45, CONT) in four consecutive harvest points; 3rd, 7th, 14th, and 60th day. Quantitative analysis showed significant differences in the directional variance of collagen fibers surrounding the scar between the NAC groups versus CONT on the 7th and 14th day. 5mm scalebar provided under each time point.

There were no statistically significant differences (ANOVA, *p* > 0.05) in the mean values of any of the variables between the NAC15, NAC30, andNAC45 groups, at all-time points. Further analyses focused on the comparison between gNAC vs. CONT (Figure 6 and Figure 7).

The density and intensity of the blue color of the collagen fibers did not vary significantly between the gNAC and the control group (*p* > 0.05) (Table 2), at any time point.

Analyzing the orientation of the collagen fibers in relation to the proximal part of the wound (P) showed that the injection of NAC at any concentration resulted in a smaller overall directional variance of collagen fibers in comparison with the control group on the 7th day (*p* = 0.02, Figure 6A). Similar observations were made on the 3rd day; however, they were not statistically significant (*p* > 0.05). On days 14 and 60, the values of the overall directional variance did not differ between the control and gNAC group. The values of all the other parameters describing the density and maturity of the fibers were similar in both compared groups.

Analysis of the scar tissue showed that the overall and local directional variances in the collagen fibers arrangement were increased in the gNAC group compared with the control group (*p* = 0.05; *p* = 0.03) on day 14 (Figure 6B). On days 3 and 7, these parameters did not vary significantly between the two groups, however, on the 60th day the variance was greater in the gNAC group, although without reaching statistical significance (*p* = 0.06). There were no differences between the studied groups in the collagen fiber density and maturity (expressed as blue color intensity) at the wound site (*p* > 0.05).

## 3. Discussion

In this study, we measured various wound/scar parameters at consecutive (11) time points. We did not observe any clinically significant side effects of enriching an anesthetic solution with NAC. Overall, out of the three analyzed concentrations of NAC the 0.03% solution had the strongest beneficial effect on wound healing. Both the area and width of the wounds were significantly reduced at the early stages of repair (days 3 and 4) in this group. The values of the wound size parameters remained the lowest in the NAC30 group compared with all the other groups throughout the observation period; however, without statistical significance. Measurements based on photography were performed at various time points, when the differences in the color of the wounds/scars could have been elusive and often difficult to recognize. Since skin in the upper or lower part of the back of animals may be stretched differently, and to avoid any bias resulting from this factor, different concentrations of NAC were used randomly at different sites (rostral, central, caudal). The most beneficial effects of NAC administration were noted at the early stages of healing, when the mean areas and widths of the wounds were lower than in the control group. These results were in accord with observations made by other researchers [12]. Oguz et al. observed a decrease in the area of scars after applying a 3% NAC ointment to the wounds.

Microscopic assessment of the wound healing process did not fully reflect the macroscopic changes we observed. At an early stage (day 3) a slightly higher number of inflammatory cells was noted (*p* > 0.05) and on the 7th day, the granulation processes were intensified (*p* = 0.06) compared with the control group. We also noted higher SCI values in the morphometrical analysis. There were no more statistically significant differences between the gNAC and CONT groups in the evaluated parameters used for subjective assessment of the wound healing. The use of Abramov’s scale with the authors’ modifications was extended for a more profound assessment of the collagen fibers. The scale was originally developed to assess excisional wound repair in rabbits [14]; though without any subsequent medical interventions, such as suturing. However, the parameters used in the scale relate to universal elements of the wound healing process, regardless of the species or the wound type. Thus, the scale was implemented in the study.

Morphometric measurements allowed illustrating the differences between the NAC30 group and the remaining groups. NAC at a 0.03% concentration significantly broadened the dermal proliferation area (DPA) and increased the scar contraction index (SCI) on the 7th day after the incision (*p* < 0.05). This outcome corresponds to a more pronounced granulation visualized during the course of the subjective histological assessment; although the latter finding did not achieve statistical significance. The scar contraction observed in a microscopic images may have reflected the smaller sizes of the scars viewed in the photographs, especially at early time points. The remaining parameters, such as the epidermis thickness, wound length, dermis thickness, or the distance between the wound margins, did not vary significantly between the control group and the NAC-treated groups. No differences in the mean values of the healing indexes were noted. Since the above-mentioned indexes were developed based on the puncture biopsy model in rabbits, their use may be limited, as the healing stages in puncture and incisional wounds are not identical [15].

An important part of wound healing is the repair of the protective barrier composed of the epidermis and dermis. The epidermis protects against abrasion and prevents an extensive loss of water or microorganism invasion, whereas elements of newly formed dermis provide mechanical strength and amortization of external physical forces acting on the epidermis [16]. Microscopic observations did not reveal significant differences in the speed of wound closure between the gNAC and the control group. Authors of similar studies have often noted a faster closure rate of wounds after NAC administration. This discrepancy may be caused by implementing different wound models in the mentioned studies. Moreover, other researchers neither sutured the wounds nor used surgical staples. The NAC route and frequency of administration, as well as the dose, differed from the methods implemented in this study [8,12,17].

In order to assess the function and structure of the repaired tissue, the organization of collagen fibers was evaluated. A manual assessment with the use of histological scales (based on the TM staining) did not reveal evident differences between the gNAC and CONT. At later time points, the directional variance of collagen fibers and their density decreased gradually, with the simultaneous increase in their maturity. The administration of NAC, however, did not affect the process significantly. By using a more precise, automated analysis of collagen fiber properties, it was possible to quantify the density and variance of the fiber directions in three compartments of each sample. NAC administration at any concentration (0.015–0.045%) resulted in a decrease in the collagen fiber directional variance at the peri-wound site on the 7th day. Furthermore, an increased directional variance of collagen fibers in the scar was noted on the 14th day, both overall and in smaller local regions. This may reflect more intensive scar remodeling. NAC did not have a significant impact on either the density or maturity of the collagen fibers. On the 60th day, no clear NAC influence on the collagen fibers was noted. To our knowledge, there have been no reports on research investigating the influence of NAC on wound healing and scar remodeling at such a late time point (60th day), and which also analyzed the collagen fiber arrangement in the newly formed skin [18].

*N*-acetylcysteine is a potent anti-oxidative substance, interacting with numerous molecular pathways, including c-Jun kinase, p38 MAP kinase, and NF kappa B transcription factor, leading to alterations of apoptosis, cellular proliferation, inflammation, endothelial function, and many more [19]. In terms of NAC’s influence on wound healing, its beneficial effect on regenerative processes was previously described [8,9,10]. Tsai et al. studied the effect of NAC in vitro and in vivo, using a burn wound healing animal model, and showed significant improvement of re-epithelialization after topical application of the substance [9]. Other studies focused on NAC’s influence on diabetic wound healing in streptozotocin- or alloxan-induced diabetes mellitus type 1 rodent models. Intraperitoneal administration of NAC decreased oxidative stress markers, as well as increased the wound-breaking strength. In the case of topical and/or systemic administration, treated groups presented smaller wound sizes (excisional model), attenuated inflammation, improved epithelialization, and fibrosis compared with the control group. The observation periods in the aforementioned studies were no longer than 14 days, thus were unable to describe the effect of NAC on the remodeling phase of wound healing. Moreover, Oguz et al. compared wound healing properties of topical NAC with well-known dexpanthenol and proved that the former was superior [12].

Choosing an optimal model for wound healing is challenging [20]. In this experiment, we used dorsal rat skin incisions to verify the influence of NAC on surgical wound healing. This design was previously used by other researcher groups, who described the impact of TGF-beta, resveratrol, and non-steroidal anti-inflammatory drugs on tissue regeneration in rats [21,22,23]. However, none of them implemented an intervention prior to wound creation.

The subtle differences in the results of histological assessment of the wound described by us and other authors may have been caused by: (1) differences in the experiment model; (2) a single NAC dose; (3) intradermal injection, a route of administration not previously described; or (4) the simultaneous injection of lidocaine and adrenaline. These substances affect the wound healing itself, as well as the distribution of a locally administered agent [24]. Further limitations concern the lack of wound-breakage assays. In summary, a beneficial effect of 0.03% NAC on wound size was observed; however, the mechanism behind this phenomenon remains unknown.

These findings require further studies on the mechanism of action of NAC administered prior to surgical incision. Exploration of the molecular impact of such an application will be further studied.

## 4. Materials and Methods

### 4.1. Animals

Animal care and handling were carried out in accordance with the UK’s Animals (Scientific Procedures) Act 1986 and associated guidelines, the EU Directive 2010/63/EU for animal experiments, and complied with the ARRIVE (Animal Research: Reporting of In Vivo Experiments) guidelines. The experiments were approved on 26.04.2017 by the First Local Ethics Committee in Warsaw (Protocol no 304/2017). The rats were purchased from the Central Laboratory of Experimental Animals of the Medical University of Warsaw (license no. 037). All surgical procedures were performed using aseptic techniques. Inbred male Sprague-Dawley rats aged 10–12 weeks (*n* = 24) were acclimatized to a 12 h light/dark cycle at 19 °C, with unlimited water and standard food. The animals were housed in a specific-pathogen-free room at the Central Laboratory of Experimental Animals, Medical University of Warsaw. The rats weighed 310 to 435 g at the time of surgery. Following surgery, the rats were housed in separate cages with environmental enrichment to avoid biting wounds by cohabitants.

### 4.2. Surgical Procedure

**Figure 8:**
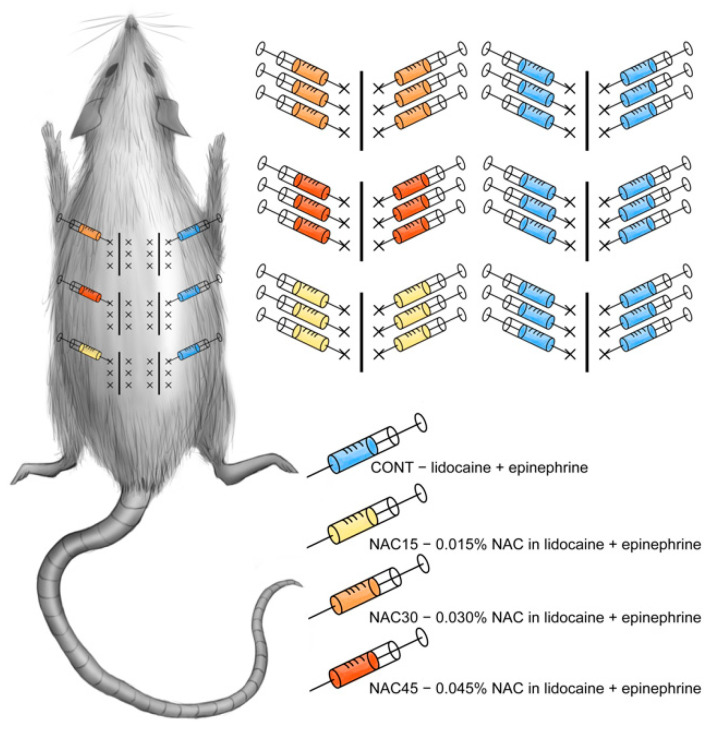
Diagram of incision lines and injection points on the rat’s dorsum. x—pre-incisional injection points of intradermal administration (0.1 mL). Straight line—marked incision line, 1.5 cm long. Two 4-0 mattress sutures were placed between the injection points of each wound.

The same anesthesia protocol was used for all animals. The rats were anesthetized with an intraperitoneal injection of ketamine (100 mg/kg bw; Ketamina, Biowet, Pulawy, Poland) and xylazine (10 mg/kg bw; Xylapan, Vetoquinol Biowet, Gorzow Wielkopolski, Poland). Each of the 24 rats had six incisions planned on the dorsal side. Incision lines were marked using a prepared permanent matrix (3 on both sides of the vertebral column), see Figure 8.

Sides (left and right) were randomly assigned to the control group (CONT) or experimental groups (NAC). One side received standard (control) solution of local anesthetic (0.5% lidocaine (Lignocainum Hydrochloricum WZF, Polfa Warsaw, Poland)) + vasoconstrictor (1:100,000 epinephrine (Adrenalina WZF, Polfa Warsaw, Poland)) 0.6 mL for each planned incision. The remaining side was treated with 3 concentrations of NAC (Acetylcysteine, Sandoz, Warsaw, Poland): 0.015%, NAC15 group, 0.03%, NAC30 group, and 0.045%, NAC45 group, dissolved in the same anesthetic solution as in the control group (0.5% lidocaine with 1:100,000 epinephrine), and randomly assigned to rostral, central, or caudal incisions.

There are scarce data on therapeutic NAC concentrations or dosing in other delivery routes than intravenous, oral, and topical administration. The dose calculation relied on safe concentrations in IV/IP/per os administrations in animal models; which ranges between 150–450 mg/kg [2,3,6], and NAC concentrations used in in vitro studies [17,25,26,27]. The drug’s bioavailability after general administration ranges from 6 to 25% [28,29]. Furthermore, we calculated the treated skin area (up to 4 cm^2^) and divided it by the rat’s total body surface (400 cm^2^ for a mass of 250 g, Meeh-Rubner formula [30]) to obtain a final concentration of 0,6ml (6 × 0.1 mL) intradermal injections-0.03% (and a 2-fold increment and decrement).

The site of each planned wound was disinfected and received six intradermal injections (Figure 8) with a 30 G needle (Sterican, B.Braun, Melsungen, Germany) 15 min prior to incising the skin. Whole thickness skin incisions (1.5 cm each) were performed with a blade no. 11 (ZARYS International Group, Zabrze, Poland). Wounds were closed with two 4-0 Prolene (Ethicon, Johnson & Johnson, New Brunswick, NJ, USA) horizontal mattress sutures. All procedures were performed by a single surgeon, blinded to the pattern of injected solutions. After surgery, the animals received an intramuscular injection of penicillin (100,000 IU/kg bw; Penicillin, Polfa, Warsaw, Poland); and intraperitoneal injections of buprenorphine chloride (0.3 μg/kg bw; Bupaq Multidose, Richter Pharma, Wels, Germany) on the first postoperative day. Sutures were removed on the 7th post-op day.

### 4.3. Evaluation of Macroscopic Wound Healing in Time; Photographic Documentation and Scar Area Quantification

**Figure 9:**
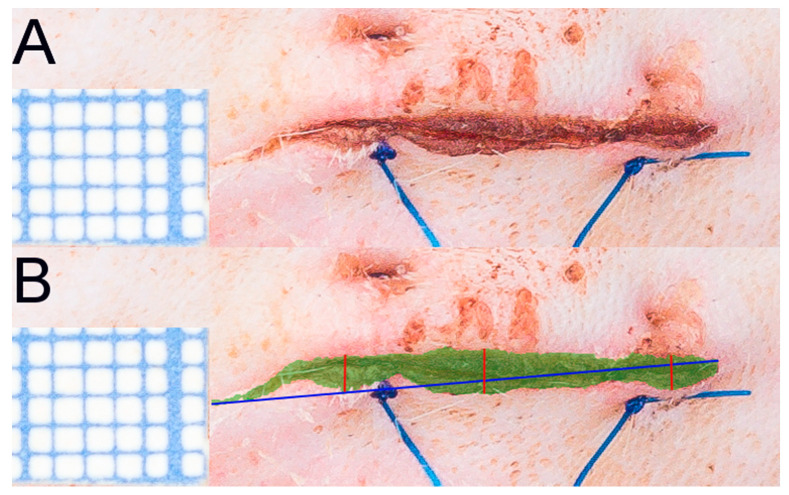
(**A**) A 3rd day wound with a surgical microscale (1 sqr–1 mm), (**B**) wound with marked area (green zone), length (blue line), and width (red lines) in ImageJ by a blinded researcher.

Standardized photographic documentation of wounds was performed on days 1, 2, 3, 4, 7, 14, 21, 28, 35, 45, and 60 after the surgery. The camera was placed on a stand 30 cm above the dorsal side of the animal. In each photo, there was a 1 cm-long millimeter-scale placed on the skin. Photos were uploaded to ImageJ 1.48 v. software (National Institutes of Health, Bethesda, MD, USA) by a blinded researcher who measured the dimensions of all scars: length (3 measures), width (3 measures at 3 regions -rostral, central, caudal part of scar), and area (3 measures) (Figure 9).

### 4.4. Scar Tissue Collection and Analysis

Groups of six rats were sacrificed on the day 3, 7, 14, or 60 after the operation. These time points were chosen to observe consecutive phases of wound healing (3rd day-inflammatory phase, 7th day-proliferative phase, 14th day-early remodeling phase, 60th day-advanced remodeling phase).

Scars were excised, divided into three equal parts, and preserved for histologic and future analyses.

The central part of the scar was preserved in 10% formalin solution for histologic and immunohistochemistry staining. Preserved specimens were prepared for paraffin embedding by an automatic tissue processor (ASP 6026, Leica, Buffalo Grove, IL, USA). Paraffin-embedded samples were sectioned to 3–5 µm slices. Sections were stained with: Hematoxylin and Eosin (HE) (Sigma-Aldrich, Saint Louis, MO, USA) in an automatic stainer (Autostainer XL, Leica, Buffalo Grove, IL, USA). Masson’s Trichrome (MT) (Sigma-Aldrich, Saint Louis, MO, USA) staining was performed to visualize the collagen fiber arrangement. Manual staining protocols were carried according to the manufacturer’s guidelines.

All stained sections were scanned at a 40× magnification in NanoZoomer XR C9600–12 (Hamamatsu, Iwata City, Japan).

### 4.5. Scar Histology Analysis

Digital scans of HE- and MT-stained scar sections underwent manual histologic assessment. Three blinded researchers performed a semi-quantitative evaluation using Abramov scale [14], specifically extended and adjusted for this study (for all rated parameters, see Supplementary Table S1). Originally, the Abramov scale produced an ordinal type of data; we employed a VAS (visual analog scale) for each parameter with 0.1 increment. The evaluation sheet was prepared online as a form with an interactive slider for each parameter, except for the complete wound closure, which was binary (www.jotform.eu, 22 March 2019).

### 4.6. Histomorphometry

**Figure 10:**
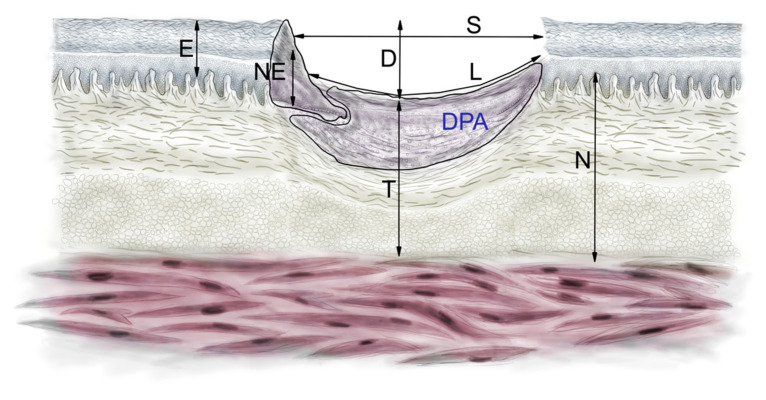
Diagram illustrates parameters measured in each HE section according to a mathematical model of wound healing proposed by Lemo et al. [15]. D—depth of the wound, DPA—dermal proliferation area, E—thickness of the epidermis, L—length of the re-epithelialization zone, N—thickness of the natural dermis, NE—thickness of the newly formed epidermis, S—distance between the borders of the wound, T—thickness of the connective tissue in the wound.

HE and MT-stained sections were also measured for critical histologic parameters describing advancement of wound healing, according to Lemo et al. [15] Dermal proliferation area (DPI) [31] was additionally implemented in this study. Measurements were taken on the digital scan of sections by a blinded researcher in QuPath v. 0.1.2 [32], Supplementary Table S2, Figure 10.

### 4.7. Collagen Fiber Arrangement Analysis

**Figure 11:**
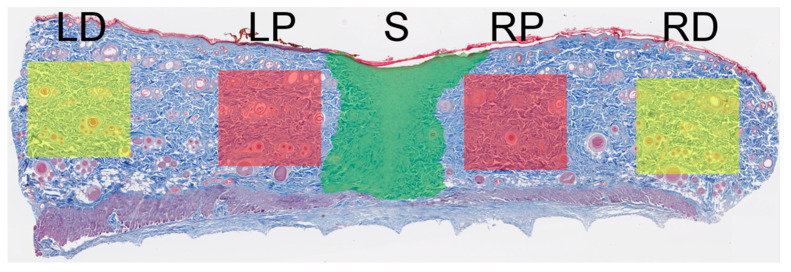
Diagram represents regions selected for automated collagen fiber analysis in MT-stained samples. Five areas were chosen, scar and four boxes 500 × 500 pixels of proximal and distant to scar area. S—scar zone, LD—left distant area, LP—left proximal area, RD—right distant area, RP—right proximal area.

Digital images of MT-stained sections were analyzed to quantify collagen fiber organization, as previously described by Quinn et al. [18,33]. Briefly, custom-written Matlab code (Mathworks, Natick, MA, USA) assessed the blue–red color intensity ratio of each pixel to determine the location of the collagen. Then the fiber orientation was calculated using a weighted vector summation approach [33]. Fiber organization was quantified as directional variance, which ranged between 0 and 1, corresponding to random and aligned fiber directions, respectively. Local directional variance measurements were assessed within a 50 pixel radius of every pixel location, while the overall directional variance measurements were assessed over the entire 500 × 500 pixel region. Four metrics were obtained: overall directional variance, average local directional variance, average fiber density, and average blue intensity. Analyses were performed within 5 regions of each sample (500 × 500 pixels for P and D, scar area individually), as described in Figure 11.

### 4.8. Statistical Analysis

Data distribution was verified with the Shapiro–Wilk test. Furthermore, two-tailed ANOVA with post-hoc Tukey test, *t*-student, and *U*-Mann–Whitney tests were used. Statistical analyses were performed in Statistica 13 (StatSoft Inc., Dell Statistica, Tulsa, OK, USA) and plots were designed in GraphPad Prism 9.1 (GraphPad Prism, San Diego, CA, USA), with the threshold of statistical significance set at *p* ≤ 0.05.

## 5. Conclusions

- Including 0.03% NAC injected intradermally prior to incision significantly decreases the area and width of a wound at an early stage of healing (3rd and 4th day) and non-significantly at later time points.
- This treatment increases the contraction and proliferation of the wound bed at an early stage. It does not affect other histologic features.
- Pre-incisional NAC mildly moderates collagen fiber orientation on the 7th and 14th day of healing and decreases variance in the scar surrounding and increases in the scar itself.

## Figures and Tables

**Table 1:** Data summarized from histomorphometrical analysis of HE- and TM-stained samples. D—depth of the wound, DPA—dermal proliferation area, E—thickness of the epidermis, L—length of the re-epithelialization zone, N—thickness of the natural dermis, NE—thickness of newly formed epidermis, S—distance between the borders of the wound, T—thickness of the connective tissue in the center of the wound, H, H0—distance between two hair follicles closest to the wound borders. H0 is the initial distance between the hair follicles (day 7), B, B0—dense scar tissue compartment, stained in blue. B0 is the total area of connective tissue in the center of the wound at day 7, SCI—superficial contraction index, DCI—deep contraction index, WSI—wound severity index, GHI—global healing index, GCI—global contraction index, HRI—hair remodeling index, MRI—matrix remodeling index, GRI—global remodeling index. Data expressed as mean ± SD; ^1^—T-test for gNAC vs. CONT, ^2^—post-hoc Tukey test for NAC30 vs. CONT. Bolded results are statistically significant.

	Day 7					Day 14			Day 60		
	CONT	gNAC	*p* ^1^	NAC30	*p* ^2^	CONT	gNAC	*p* ^1^	CONT	gNAC	*p* ^1^
D (µm)	527.2 ± 279.3	627.5 ± 193.2	0.34	558.3 ± 251.7	0.86	313.0 ± 96.8	375.8 ± 191.8	0.76	157.2 ± 98.2	143.4 ± 88.0	0.67
DPA (µm^2^)	134,280.2 ± 60364.62	180,923.8 ± 190501.1	0.46	353,591.2 ± 314148.8	**0.04**	405,838.2 ± 226281.4	550,007.5 ± 508,475.6	0.27	906,748.8 ± 572,360.8	743,650.1 ± 336,697.1	0.30
EPI (µm)	50.9 ± 13.1	43.3 ± 11.5	0.17	42.4 ± 12.2	0.34	44.4 ± 10.2	46.0 ± 14.3	0.71	28.1 ± 6.04	28.4 ± 5.21	0.85
L(µm)	1909.8 ± 901.6	1932.1 ± 649.9	0.94	1471.0 ± 810.9	0.46	1272.9 ± 484.5	1382.7 ± 570.5	0.58	793.99 ± 456.6	919.22 ± 815.1	0.59
N (µm)	1434.4 ± 196.7	1427.2 ± 169.5	0.92	1459.5 ± 65.5	0.83	1404.1 ± 187.8	1496.0 ± 393.1	0.96	1680.7 ± 369.3	1625.0 ± 246.4	0.60
NEO (µm)	83.6 ± 23.4	98.8 ± 24.4	0.16	102.3 ± 43.85	0.33	110.1 ± 28.9	114.2 ± 33.2	0.72	38.1 ± 7.82	37.3 ± 12.9	0.82
S (µm)	1533.2 ± 791.5	1312.6 ± 630.3	0.48	820.3 ± 670.0	0.18	1150.8 ± 541.2	1268.9 ± 676.7	0.61	696.69 ± 403.1	836.99 ± 776.4	0.52
T (µm)	915.5 ± 298.8	802.9 ± 270.5	0.37	794.5 ± 145.9	0.52	1289 ± 192.6	1484.3 ± 381.8	0.21	1650.1 ± 435.3	1542.3 ± 275.6	0.40
H (µm)	1604.5 ± 980.1	1448.4 ± 1152.6	0.66	1731.1 ± 762.5	0.79	1890.3 ± 1082.3	1679.2 ± 1134.4	0.57	542.48 ± 248.6	736.08 ± 705.2	0.27
B (µm^2^)	189,079.1 ± 127,604.5	155,889.7 ± 140,034.4	0.46	222,271.6 ± 196,386.6	0.65	652,444.9 ± 293,520.0	622,769.1 ± 387,104.4	0.79	619,803.1 ± 407,497.9	645,390.3 ± 409,208.7	0.85
SCI	0.21 ± 0.15	0.34 ± 0.15	0.07	0.46 ± 0.18	**0.03**	0.11 ± 0.21	0.11 ± 0.25	0.94	0.12 ± 0.08	0.10 ± 0.07	0.44
DCI	0.63 ± 0.15	0.55 ± 0.13	0.22	0.62 ± 0.16	0.86	0.77 ± 0.06	0.77 ± 0.07	0.84	0.90 ± 0.06	0.91 ± 0.05	0.58
WSI	0.35 ± 0.22	0.44 ± 0.14	0.27	0.45 ± 0.12	0.48	0.09 ± 0.14	0.04 ± 0.17	0.40	0.01 ± 0.11	0.05 ± 0.10	0.34
GHI	0.49 ± 0.32	0.45 ± 0.31	0.76	0.63 ± 0.39	0.55	0.80 ± 0.22	0.84 ± 0.30	0.69	1.01 ± 0.18	0.96 ± 0.15	0.42
GCI	0.85 ± 0.21	0.90 ± 0.21	0.59	1.08 ± 0.27	0.13	0.89 ± 0.22	0.88 ± 0.28	0.90	1.02 ± 0.10	1.01 ± 0.09	0.75
RHI	−2.76 ± 0.61	0.09 ± 0.71	0.66	−0.07 ± 0.47	0.79	−0.17 ± 0.67	−0.04 ± 0.70	0.57	0.66 ± 0.15	0.54 ± 0.43	0.27
MRI	2.35 ± 0.67	0.17 ± 0.74	0.46	−0.17 ± 1.03	0.65	−2.45 ± 1.55	−2.29 ± 2.04	0.79	−2.27 ± 2.15	−2.41 ± 2.16	0.85
GRI	−2.09 ± 0.44	0.13 ± 0.61	0.45	−0.12 ± 0.51	0.58	−1.31 ± 1.06	−1.17 ± 1.02	0.68	−0.80 ± 1.11	−0.93 ± 1.22	0.74

**Table 2:** Results obtained from automated analysis of collagen fiber setting on TM-stained sections. Values expressed as mean ± SD; ^1^—*t*-test for gNAC vs. CONT. Bolded results are statistically significant.

Day 3									
	Distal Areas (RD&LD) (*n* = 36)			Proximal Areas (RP&LP) (*n* = 36)			Scar Area (S) (*n* = 18)		
	CONT	gNAC	*p* ^1^	CONT	gNAC	*p* ^1^	CONT	gNAC	*p* ^1^
Directional Variance	0.86 ± 0.05	0.85 ± 0.05	>0.05	0.84 ± 0.06	0.82 ± 0.07	>0.05	0.88 ± 0.05	0.89 ± 0.05	>0.05
Local Directional Variance	0.78 ± 0.03	0.79 ± 0.04	>0.05	0.78 ± 0.03	0.76 ± 0.05	>0.05	0.76 ± 0.04	0.76 ± 0.02	>0.05
Fiber Density	0.74 ± 0.07	0.74 ± 0.07	>0.05	0.74 ± 0.06	0.75 ± 0.07	>0.05	0.62 ± 0.09	0.60 ± 0.08	>0.05
Blue Intensity	0.71 ± 0.03	0.71 ± 0.04	>0.05	0.71 ± 0.02	0.71 ± 0.03	>0.05	0.72 ± 0.02	0.71 ± 0.02	>0.05
**Day 7**									
Directional Variance	0.88 ± 0.06	0.87 ± 0.07	>0.05	0.88 ± 0.05	0.85 ± 0.05	**0.02**	0.92 ± 0.03	0.90 ± 0.04	>0.05
Local Directional Variance	0.78 ± 0.03	0.78 ± 0.04	>0.05	0.79 ± 0.03	0.77 ± 0.03	>0.05	0.76 ± 0.02	0.76 ± 0.02	>0.05
Fiber Density	0.73 ± 0.05	0.75 ± 0.05	>0.05	0.75 ± 0.08	0.74 ± 0.07	>0.05	0.62 ± 0.07	0.63 ± 0.08	>0.05
Blue Intensity	0.71 ± 0.03	0.71 ± 0.03	>0.05	0.71 ± 0.03	0.71 ± 0.03	>0.05	0.72 ± 0.02	0.72 ± 0.03	>0.05
**Day 14**									
Directional Variance	0.86 ± 0.06	0.88 ± 0.07	>0.05	0.86 ± 0.09	0.87 ± 0.05	>0.05	0.87 ± 0.07	0.92 ± 0.03	**0.05**
Local Directional Variance	0.76 ± 0.04	0.77 ± 0.03	>0.05	0.76 ± 0.06	0.76 ± 0.03	>0.05	0.74 ± 0.03	0.76 ± 0.02	**0.03**
Fiber Density	0.69 ± 0.15	0.72 ± 0.10	>0.05	0.68 ± 0.16	0.73 ± 0.09	>0.05	0.61 ± 0.09	0.64 ± 0.13	>0.05
Blue Intensity	0.76 ± 0.05	0.75 ± 0.04	>0.05	0.76 ± 0.05	0.75 ± 0.05	>0.05	0.76 ± 0.05	0.76 ± 0.04	>0.05
**Day 60**									
Directional Variance	0.86 ± 0.06	0.86 ± 0.06	>0.05	0.84 ± 0.05	0.85 ± 0.05	>0.05	0.86 ± 0.03	0.89 ± 0.05	>0.05
Local Directional Variance	0.77 ± 0.04	0.77 ± 0.04	>0.05	0.76 ± 0.03	0.76 ± 0.03	>0.05	0.75 ± 0.02	0.77 ± 0.03	>0.05
Fiber Density	0.73 ± 0.06	0.74 ± 0.03	>0.05	0.74 ± 0.06	0.72 ± 0.05	>0.05	0.75 ± 0.05	0.74 ± 0.06	>0.05
Blue Intensity	0.71 ± 0.03	0.71 ± 0.04	>0.05	0.71 ± 0.02	0.71 ± 0.03	>0.05	0.72 ± 0.02	0.71 ± 0.02	>0.05

## Data Availability

The data that support the findings of this study are available from the corresponding author, WP, upon reasonable request.

## Supplementary Materials

The following are available online at https://www.mdpi.com/article/10.3390/ijms22147549/s1, Supplementary Table S1: Modified Abramov scale for histological skin wound healing assessment Table S2: Definition and measurement technique of wound healing parameters and indexes.
