# Supplementary material for: N-Acetylcysteine Added to Local Anesthesia Reduces Scar Area and Width in Early Wound Healing—An Animal Model Study
Source: Int J Mol Sci. 2021 Jul 14;22(14):7549. doi: 10.3390/ijms22147549 (PMC8307704; doi:10.3390/ijms22147549)
Supplement: Supplementary file 1 [file ijms-22-07549-s001.zip › ijms-1250809-supplementary.pdf]

Supplementary materials

Table S1–Modified Abramov scale for histological skin wound healing assessment.

HE Staining	VAS Scale (0.1 increment)			
Parameter	0	1	2	3
Acute inflammation	None	Scant	Moderate	Abundant
Chronic inflammation	None	Scant	Moderate	Abundant
Granulation tissue amount	None	Scant	Moderate	Abundant
Granulation tissue fibroblast maturation	Immature	Mild maturation	Moderate maturation	Fully maturated
Collagen deposition	None	Scant	Moderate	Abundant
Reepithelialization	None	Partial	Complete but immature or thin	Complete and mature
Neovascularization	None	Up to 5 vessels per HPF	6-10 vessels per HPF	>10 vessels per HPF
	Binary			
Complete wound closure	0		1	
**TM Staining**	**VAS Scale (0.1 increment)**			
Parameter	0		10	
Collagen fiber orientation regularity	Uniform/regular		Chatoic/irregular	
Collagen fiber density	Homogenous		Heterogenous	
Collagen fiber diameter	Uniform/regular		Variable/irregular	
Colagen fiber maturity	Old/mature fibers		Newly formed collagen fibers	
Overall assessment of collagen fiber setting	Unaffected collagen fiber setting - correspond to a normal skin section. Regular fiber orientation, uniform fiber diameter		Chaotic collagen fiber distribution, deposits of a new collagen, variable fiber orientation, diameter and density	
	0	1	2	3
Granulation tissue amount	None	Scant	Moderate	Abundant
Collagen deposition	None	Scant	Moderate	Abundant

Table S2–Definition and measurement technique of wound healing parameters and indexes

Parameter	Definition	Tool and Measurement Technique
	**HE Staining**	
E	The thickness of the epithelium at least 1mm away from both edges of the wound	5 measurements on both sides of the wound with the line tool, set perpendicularly from stratum corneum to stratum basale.
NE	The thickness of newly formed epithelium over the scar	6 measurements with the line tool, set perpendicularly from stratum corneum to stratum basale
S	The distance between the borders of the wound, following the straight line of epidermis.	3 measurements – stright lines (line tool) between the wound edges
L	The length of the reepithelialization zone, between the borders of the wound.	3 measurements with polygonal tools – surface between wound edes lying on the granulation tissue/stratum basale of the epithelium

D	The depth of the wound, from the epidermis line (S line) to the first connective tissue layer at the deepest point of the wound	3 measurements with the line tool – line starts perpendicular do line S and ends in the deepest point of the wound. If unable to set – left as "0"
T	The thickness of the connective tissue in the centre of the wound , from the deepest point of the wound to the muscle	3 measurements with the line tool – line starts in the deepest point of the wound and runs down to the end of subcutaneous tissue. If unable to set – left as "0"
N	The thickness of the natural dermis on both sides of the wound, from the muscle to the epidermis	6 measurements with the line tool, set perpendicularly from stratum corneum to stratum basale (3 per each side of the wound)
DPA	Dermal proliferation area	3 measurements with the polygonal tool under the wound surface. Measurements contain granulation tissue without inflammatory influx.
	**TM Staining**	
H, H_0	The distance between the first hairs on each border of the wound. H_0 is the initial distance between the first hairs (day 7)	3 measurements with the line tool between first hair follicles of the wound edges
B, B_0	Stained in blue and corresponds to a dense scar tissue. B_0 is the total surface of wounded connective tissue in the centre of the wound at day 7	3 measurements with the polygonal tool containing connective tissue of the scar without epithelium.
	**Wound Healing Indexes**	
SCI	Superficial contraction index	$\mathrm{SCI} = \frac{\mathrm{L} - \mathrm{S}}{\mathrm{L}}$
DCI	Deep contraction index	$\mathrm{DCI} = \frac{N - D}{\mathrm{N}}$
WSI	Wound severity index	$\mathrm{WSI} = \frac{\mathrm{N} - \mathrm{T}}{\mathrm{N}}$
GHI	Global healing index	$\mathrm{GHI} = \mathrm{SCI} + \mathrm{DCI} - \mathrm{WSI}$
GCI	Global contraction index	$\mathrm{GCI} = \mathrm{SCI} + \mathrm{DCI}$
HRI	Hair Remodelling Index	$\mathrm{HRI} = \frac{H_0 - \mathrm{H}}{H_0}$
MRI	Matrix Remodelling Index	$\mathrm{MRI} = \frac{B_0 - \mathrm{B}}{B_0}$
GRI	Global Remodelling Index	$\mathrm{GRI} = \frac{\mathrm{HRI} + \mathrm{MRI}}{2}$
